# Preparation of Chlorpheniramine Maleate-loaded Alginate/Chitosan Particulate Systems by the Ionic Gelation Method for Taste Masking

**DOI:** 10.17795/jjnpp-12530

**Published:** 2014-02-20

**Authors:** Mitra Jelvehgari, Leila Barghi, Farhad Barghi

**Affiliations:** 1 Drug Applied Research Center, Tabriz University of Medical Sciences, Tabriz, IR Iran; 2 Faculty of Pharmacy, Tabriz University of Medical Sciences, Tabriz, IR Iran; 3 Student Research committee, Tabriz University of Medical Sciences, Tabriz, IR Iran

**Keywords:** Chlorpheniramine, Calcium, Aluminium, Taste

## Abstract

**Background:**

Chlorpheniramine maleate (CM) is widely used as an antihistaminic drug but it is very bitter and as yet no mouth dissolving/disintegrating taste-masked preparation that might be useful for pediatric and geriatric patients is available in the market.

**Objectives:**

The purpose of this research was to mask the bitter taste of CM by formulating microspheres of the taste-masked drug.

**Materials and Methods:**

This work was done to develop alginate/chitosan particles prepared by ionic gelation (Ca^2+^ and Al^3+^) for the CM release. The effect of different chitosan and Ca^2+^ concentrations on taste masking and the characteristics of the microspheres were investigated. Ca^2+^ and Al^3+^ alginates microspheres of CM were prepared using cross-linked insoluble complexes that precipitate, incorporating the drug. Formulations were characterized for particle size and shape, entrapment efficiency, fourier transform spectroscopy (FTIR), x-ray diffraction (XRD), and differential scanning calorimetry (DSC), bitter taste threshold and *in vitro* drug release in simulated gastrointestinal fluids.

**Results:**

FTIR, XRD and DSC demonstrated unstable characters of CM in the drug-loaded microspheres and revealed an amorphous form. Also, the peak of alginate microparticles (Ca^2+^ and Al^3+^ ions) in all formulations remained the same, with low intensity of spectrum. The results of DSC, X-ray diffraction and FTIR showed the presence of several CM chemical interactions with alginate and ions (Ca^2+^ and Al^3+^). The microsphere formulations showed desirable drug entrapment efficiencies (62.2-94.2%). Calcium/aluminum alginate retarded the release of CM at low pH = 1.2 and released the drug from microspheres slowly at pH = 6.8, simulating intestine pH. The drug release duration and the release kinetics were dependent on the nature of the polymers, the cation concentrations, and valences (Ca^2+^ and Al^3+^). The drug release rate was decreased by an increase in chitosan and cation concentrations.

**Conclusions:**

The results of the present study indicated that oral preparation of CM with an acceptable taste is feasible.

## 1. Background

In recent years, biodegradable polymers have attracted attention as biomaterials particularly for tissue engineering, gene therapy, wound healing and controlled drug delivery systems (1). Chitosan being a cationic polymer has been used for the production of microspheres and nanoparticles by ionotropic gelation with negatively charged polymers and there are many chitosan-polyanion complexes that have been investigated as drug delivery systems for drugs (2). Alginates are random, linear and anionic polysaccharides consisting of linear copolymers of a-L-guluronate and b-D-mannuronate residues (3-5). Among polyanionic polymers, alginate has been widely studied and applied for its potential to modulate drug release, and its biodegradability and lack of toxicity (6, 7). Chitosan-alginate polyionic complexes are formed through ionic gelation via interactions between the carboxyl groups of alginate and the amine groups of chitosan.

Bitter taste of CM is felt when it comes in direct contact with mucous membranes of the tongue and taste buds, which reduce patient compliances causing the patients to feel unsatisfied. Taste masking of the drug as microparticles by the ionotropic gelation technique effect its release profile and other properties. In addition, chemical methods (after electrostatic reaction of cations with anions) such as altering the chemical structure of the drug itself have been used to remove the bitter taste (8).

## 2. Objectives

The objective of the present work is to develop an efficient method to mask the bitter taste of CM via microparticles using the ionotropic gelation technique.

## 3. Materials and Methods

### 3.1. Material

Sodium alginate (low viscosity, 50 mPa-s (millipascal-second) and low-molecular weight chitosan (20-200 centi-poise viscosity) with a deacetylation degree of 75-85% were purchased from Sigma-Aldrich (Germany), cholorpheniramine was purchased from Pingguang Pharmaceutical (China). All solvents and reagents were obtained from Merck (Darmstadt, Germany).

### 3.2. Preparation of Microspheres

All microspheres were obtained by the ionotropic gelation method using Ca^2+^ or Al^3+^ ions. Primarily, CM (0.25% w/v) was added to an aqueous solution of alginate (1.2% w/v) and agitated continuously on a magnetic stirrer until complete dissolution. Next, chitosan was dissolved in glacial acetic solution (0.5% v/v) to which Ca^2+^ (or Al^3+^) ions (1.3% w/v) were added and mixed using a magnetic stirrer. The solution containing alginate and CM was then injected into the chitosan solution using a hypodermic needle. Microspheres formed rapidly and were left in the solution for 24 hours to ensure internal gelification. Finally, microspheres were filtered, washed and dried at room temperature (Tables 1 and 2). F4, F5, F6 (Ca^2+^) and F’4 (Al^3+^) particles (without chitosan) were prepared under the same conditions. Morphology of CM microspheres was determined by using an optical microscope (Nikon, Japan).

**Table 1.** Chlorpheniramine Maleate Microparticle Formulations Prepared by the Ionotropic Gelation Method With Different Sodium Alginate:Chitosan Ratios, Without Chitosan and Ion (Ca^2+^) ^a^

Formulations	Na-Alg:CS Ratio	Cross-linked Insoluble Complexes					
		Aqueous Phase			Ionotropic Gelation Agents		
		CM, g	Water, mL	Na-Alg, g	Chitosan, g	Acetic Solution (%0.5 v/v), mL	Calcium Chloride, g
**F1**	1:0.27	0.25	100	1.2	0.325	100	1.3
**F2**	1:0.54	0.25	100	1.2	0.650	100	1.3
**F3**	1:1.1	0.25	100	1.2	1.3	100	1.3
**F4**		0.25	100	1.2		100	0.65
**F5**		0.25	100	1.2		100	1.3
**F6**		0.25	100	1.2		100	2.6

^a^ Abbreviations: Na-Alg, sodium alginate; CS: chitosan; CM, chlorpheniramine.

**Table 2.** Chlorpheniramine Maleate Microparticle Formulations Prepared by the Ionotropic Gelation Method With Different Sodium Alginate:Chitosan Ratios, Without Chitosan and Ions (Al^3+^) ^a^

Formulations	Na-Alg:CS Ratio	Cross-linked Insoluble Complexes					
		Aqueous Phase			Aqueous Phase		
		Chlorpheniramine, g	Water, mL	Na-Alg, g	Chitosan, g	Acetic Solution (%0.5 v/v), mL	Aluminum Chloride, g
**F’1**	1 :0.27	0.25	100	1.2	0.325	100	1.3
**F’2**	1:0.54	0.25	100	1.2	0.650	100	1.3
**F’3**	1:1.1	0.25	100	1.2	1.3	100	1.3
**F’4**		0.25	100	1.2		100	1.3

^a^ Abbreviations: Na-Alg, sodium alginate; CS, chitosan.

### 3.3. Determination of Drug Load and Entrapment Efficiency

The drug concentration was determined spectrophotometrically (UV-160, Shimadzu, Japan) at 261.8 nm by measuring the amount of non-entrapped CM in the external aqueous solution (indirect method) (9). CM entrapment efficiency was expressed as the ratio of CM measured in the supernatant to the total CM added (9).

### 3.4. Particle size Analysis

The microsphere size analysis was performed using laser light scattering particle size analyzer (SALD-2101, Shimadzu, Japan).

### 3.5. Differential Scanning Calorimetery

Samples of the microparticles (about 5 mg) were heated (5-300˚C) at a scanning rate of 10˚C/min in crimped sealed aluminum pans under a nitrogen atmosphere (DSC 60, Shimadzu, Japan).

### 3.6. X-ray Powder Diffractometry

X-ray diffraction analysis was performed with an apparatus (Siemens D5000, Munich, Germany), using nickel-filtered CuKα radiation (a voltage of 40 KV and a current of 20 mA). The scanning rate was 2˚C/min over a range of 20-70˚C and with an interval of 0.02˚C.

### 3.7. Fourier Transforms Infrared Spectroscopy

A computerized Fourier, which transforms infrared spectroscopy, FT-IR (Bomen, Quebec, Canada) was used to obtain the spectra of various CM samples. The scanning range was 400-4000 cm^-1^ and the resolution was 1 cm^-1^.

### 3.8. Assessment of the Bitter Taste of Microspheres

#### 3.8.1. Standard Solution for Evaluation of the Bitter Taste Threshold of CM

The bitter taste threshold value of CM was determined based on the bitter taste recognized by 8 volunteers (4 females and 4 males). A series of CM aqueous solutions were prepared at different concentrations as standard solutions. The test was performed as follows: 1 mL of each standard solution was placed on the center of the tongue, it was retained in the mouth for 30 seconds, and then the mouth was thoroughly rinsed with distilled water. The threshold value was correspondingly selected from different CM concentrations as the lowest concentration that had a bitter taste. Microspheres of CM (10 mg) were put into 10 mL distilled water. The mixture was immediately vibrated for 30 seconds and then filtered. Then the solution was analyzed spectrophotometricaly at 261.8 nm to determine the dissolved drug concentration in water, which was then compared with the threshold value (10).

### 3.9. *In Vitro* Release Studies

The *in vitro* release studies of drug-loaded microspheres were carried out at 37˚C in acidic conditions (pH = 1.2) for 2 hours followed by 6 hours dissolution in 0.2 M phosphate buffer by the basket method (pH = 6.8). After 2 hours, 17 mL of 0.2 M phosphate buffer stock, pre-equilibrated at 37˚C, was added to the dissolution vessel. The pH was immediately adjusted, if necessary, with 0.2 N HCl or 0.2 N (normal) NaOH to pH = 6.8. Each batch of microspheres containing 4 mg of drug was individually added to 900 mL of dissolution medium (RPM = 100) in a flask. After suitable dilution (concentration correction), the drug from each sample at pH = 1.2 and 6.8 was estimated using UV spectrophotometer analysis at 264.6 and 261.6 nm, respectively. Each experiment was repeated three times. The sample (5 mL) was withdrawn at 5, 15, 30, 60, 120, 180, 240, 300, 360 and 480 minutes intervals and replaced with the same volume of test medium and the withdrawn samples were diluted if required and then analysed using UV spectrophotometer analysis at 264.6 and 261.6 nm, respectively (UV-160, Shimadzu, Japan). Dissolution efficiency (DE), t_50_% (dissolution time for 50% fraction of drug), and difference factor, f1 (used to compare multipoint dissolution profiles) are calculated order to have a better comparison between different formulations and the results are listed in Table 3 (11).

**Table 3.** Effect of Sodium Alginate:Chitosan Ratio and Ions (Ca^2+^, Al^3+^) on the Content, Production Yield and Particle Size of Chlorpheniramine Microparticles

Form-ulations	Chitosan, g	Na-Alg:Cs Ratio	Production Yield, % (SD)	Theoretical Drug Content, %	Mean Amount of Drug Entrapped, % (SD)	Loading Efficiency , % (SD)	Mean Particlesize, µm (SD)
**F1**	0.325	1 :0.27	40 (3.65)	8.13	1.7 (0.27)	20.91 (3.78)	644.54 (59.15)
**F2**	0.65	1:0.54	49.41 (5.69)	7.35	2.32 (0.34)	31.56 (2.89)	728.29 (48.27)
**F3**	1.3	1:1.1	51.6 (6.31)	6.17	3.84 (0.65)	62.24 (3.62	769.09 (41.37
**F4**			55.45 (5.63)	11.90	11.21 (3.85)	94.20 (7.48	731.62 (39.96)
**F5**			58.18 (6.39)	9.09	6.17 (1.12)	67.88 (4.69)	1052.44 (57.73)
**F6**			53.09 (4.32)	6.17	5.09 (1.47)	82.5 (7.25)	1061 (45.27)
**F’1**	0.325	1 :0.27	46.83 (4.12	8.13	6.19 (0.65)	76.14 (6.25)	2251.85 (11.96)
**F’2**	0.65	1:0.54	49.41 (4.89)	7.35	5.92 (0.47)	80.54 (6.89)	1288.25 (11.79)
**F’3**	1.3	1:1.1	45.43 (4.28)	6.17	2.95 (0.26)	47.81 (5.45)	1513.56 (10.22)
**F’4**			44 (5.91)	9.09	9.77 (2.11)	107.48 (8.19)	912.01 (11.79)

DE is defined as the area under the dissolution curve up to a certain time, *t*, expressed as a percentage of the area of the rectangle arising from 100% dissolution during the same time. The areas under the curve (AUC) were calculated for each dissolution profile by the trapezoidal rule. DE can be calculated by the following equation 1:

Equation 1. DE = ʃy dt/100t

Where, y is the drug percent dissolved at time *t*. The *in vitro* release profiles of different microparticle formulations were compared with physical mixture formulations using difference factor (*f*1), as defined by Equation 2 (11):

**Equation 2:**
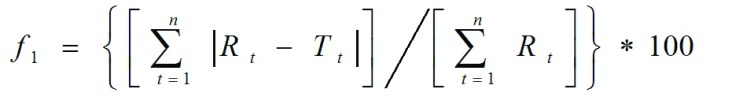


Where, n is the number of time points, R_t_ is the dissolution value of the reference batch at time t, and T_t_ is the dissolution value of the test batch at time t. Data obtained from *in vitro* release studies were fitted to various kinetic equations (zero, first, Higuchi model).

### 3.10. Statistical Analysis

Results were evaluated using a one-way ANOVA (SPSS version 14), where P < 0.05 was taken to represent a statistically significant difference.

## 4. Results

### 4.1. Microsphere Characterization

**Figure 1.**
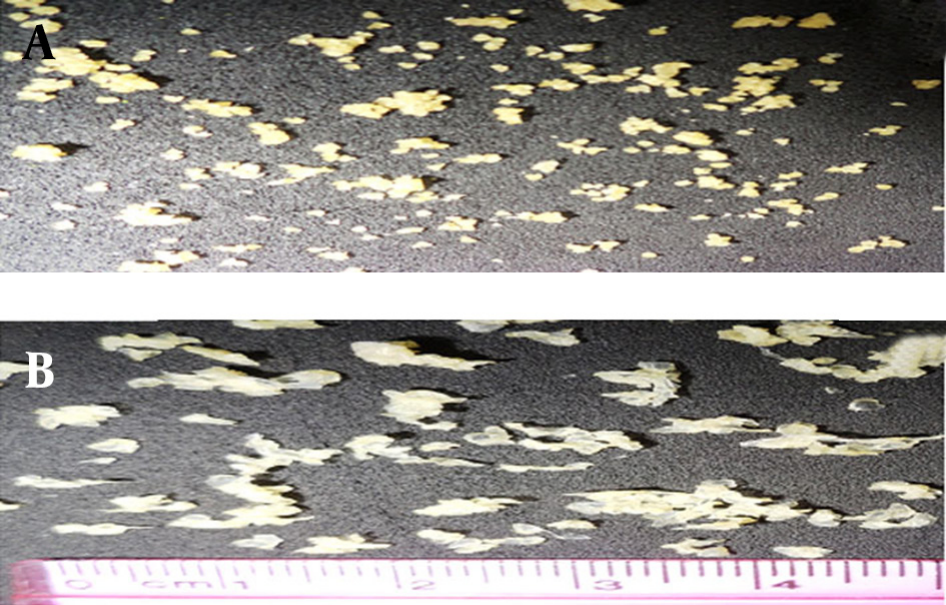
Optical Microscopic Photograph of Spherical Microspheres A. F3 (1.3 g chitosan and 1.3 g calcium chloride) and B. F5 (0.325 g chitosan and 1.3 g aluminum chloride) formulations at 10x.

In this paper we evaluate the potential utility of natural materials, such as alginate and chitosan for taste masking of CM. This microparticulate system extends the duration period of the drug and masks the bitter taste of the drug. Alginate/chitosan particles were prepared by ionic gelation (Ca^2+^ and Al^3+^) and the effects of various factors (including chitosan and ions concentration) were examined. The ionic gelation method gave beads with a large diameter ranging from 644-1061 µm (Ca^2+^) and 912-2251 µm (Al^3+^) (Table 3). The drug encapsulation efficiency was 20.9 to 62.2% for Ca^2+^ ions with chitosan, 67.88 to 94.20% without chitosan and 47.8 to 76.1% for Al^3+^ ion with chitosan, 107.4%, without chitosan, respectively. Alginate-chitosan gels produced the egg-box structure of the beads, which gives it the spherical morphology. Figure 1A shows a microsphere prepared from adding Ca^2+^; exhibiting acceptable sphericity in all cases. The beads were off-white, transparent and elastic (Figure 1A and B).

The aspect and morphology of particulates prepared with Al^3+^ ions is different. None of the formulations resulted in the formation of a spherical morphology; on the contrary, the particles were flattened, disc-shaped with a collapsed center. (Figure 1B). The production yield was 40-51.6% for Ca^2+^ ion with chitosan and 53.1-58.2%, without chitosan. The production yield was 45.4-49.4%, for Al^3+^ ions with chitosan and 44%, without chitosan.

### 4.2. DSC, XRD and FTIR Studies

**Figure 2.**
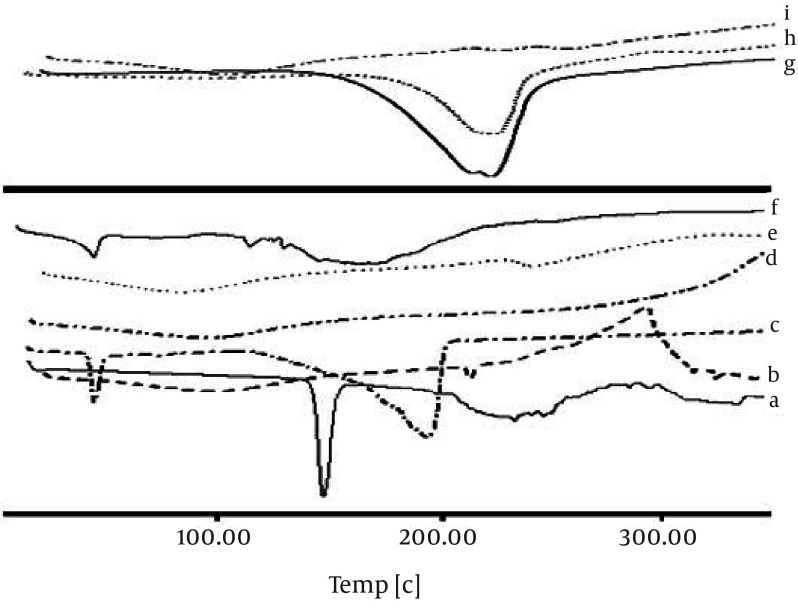
a. DSC Thermogram of Chlorpheniramine; b. Na alginate; c. Calcium chloride; d. Chitosan; e. F3; f. Physical Mixture F3; g. Aluminum chloride; h. Physical Mixture F’1; i. F’1

Any abrupt or drastic change in the thermal behavior of either the drug or the polymer may indicate a possible drug-polymer interaction. The DSC curve of the CM (Figure 2) shows an endothermic peak at 134.78˚C, which cor responds to its melting point. However in the thermogram of the microparticles, the endothermic peak corresponding to the drug melting was absent, suggesting the amorphous state of the drug in the microparticles.

**Figure 3.**
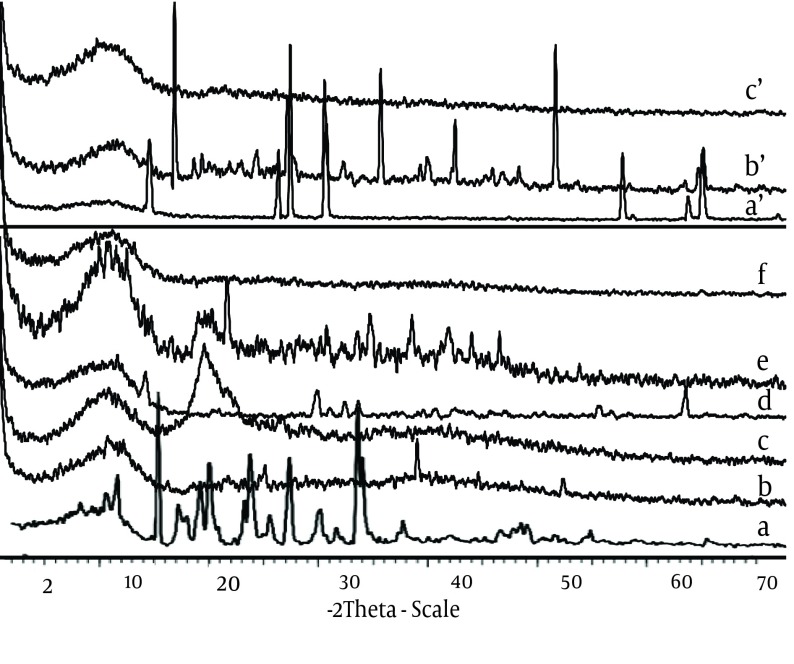
a. X-ray Diffraction of Chlorpheniramine; b. Na alginate; c. Chitosan; d. Calcium chloride; e. Physical Mixture F3, f. F3; aʹ. Aluminum chloride; bʹ. Physical Mixture F’1; cʹ. F’1.

CaCl_2_ shows two endothermic peaks at 48.23 (absorb or adsorb of water) and 174.31˚C (water of hydration) and AlCl_3_ at 192˚C; while alginate decomposes at about 240˚C with a broad exothermic peak. Physical mixture of F3 and F1 microparticles (Ca^2+^ and Al^3+^ ions) shows only an endothermic peak of Ca^2+^ and Al^3+^ ions, respectively. The X-ray diffraction patterns show that the pure drug is crystalline in nature (Figure 3). However, when it was incorporated into the polymer matrix, the principal peaks of the drug disappeared.

**Figure 4.**
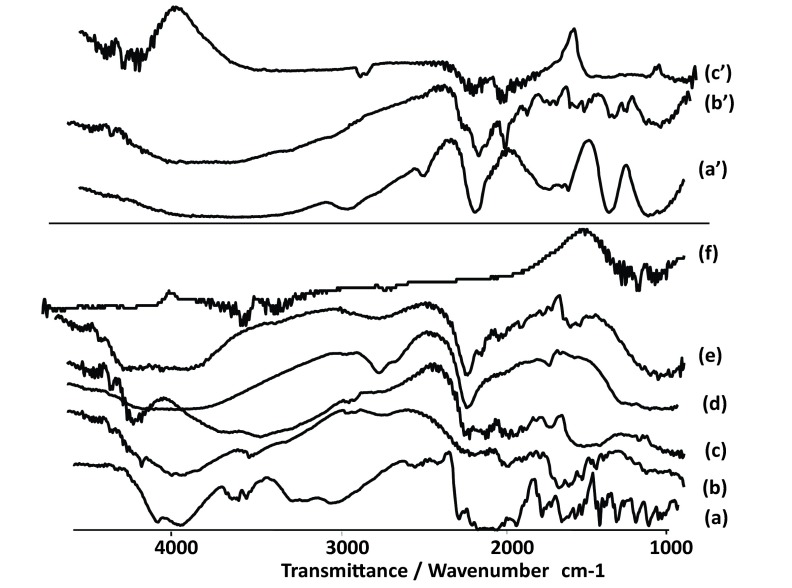
a. FTIR Spectrum of Chlorpheniramin; b. Na Alginat; c. Chitosan; d. Calcium Chloride; e. Physical Mixture F3, f. F3 (f); aʹ. Aluminum Chloride; bʹ. Physical Mixture F’1; cʹ. F’1

The FT-IR spectra of pure CM (Figure 4) depicts three characteristic bands at 1580 cm^-1^, 1476 cm^-1^ and 1352 cm^-1^ due to C=C stretching, C-H stretching and C-H bending, respectively. Another two sharp bands can be seen at 864 cm^-1^ and 702 cm^-1^, which are due to C-C and C-Cl stretching vibration.

In the spectrum of Alg and chitosan (Figure 4) the band at 3318 cm^-1^ is for stretching vibration of OH of COOH group, 3277 cm-1 for stretching vibration of NH group, 1716 cm^-1^ for stretching vibration of C=O attached to the methylene group and ether, 1278 cm^-1^ for C-N stretching vibration of secondary aromatic amine, 1151 cm^-1^ for aliphatic ether (asymmetric C-O-C stretching), and the band at 1052 cm^-1^ is for OH bending vibration. Chitosan and alginate functional group’s peaks shifted after ionic interaction. The microspheres exhibited the characteristic peaks of C=O stretching band at 1420 cm^-1^ (Figure 3). Other peaks present symmetric stretching of the carboxyl group at 1610 cm^-1^, 1616 cm^-1^ and 1534 cm^-1^. Also symmetric stretching of the carboxyl group is indicated at 1414 cm^-1^. The characteristic OH stretching, NH stretching, C-H stretching and C=O stretching of pure drug was changed in the spectra of the microspheres.

### 4.3. The bitter Taste Studies

Bitter drugs are entrapped in the polymers, thereby offering a barrier between the drug and the taste receptors of the tongue. As a result the drug cannot bind with the taste receptors and therefore the taste is not sensed (12, 13).

### 4.4. Determination of Bitter Recognition Threshold of CM

All eight volunteers could not recognize the bitter taste of CM at 50 μg/mL. Five out of eight volunteers could perceive the bitter taste at 100 μg/mL, whereas all eight volunteers reported that the solutions of 100 and 150 μg/mL were bitter. Thus, the bitterness threshold value lies between 100 to 150 μg/mL. Therefore, solutions of the CM at concentrations of 50, 100, 150, 250, 350 and 450μg/mL were prepared and the same procedure was repeated. From Table 4 it can be figured that the bitter taste threshold value of CM is around 100 μg/mL. Concentration of drug released from all the polymeric microspheres was lower the bitter threshold of CM. The results are shown in Table 5. 

**Table 4.** Taste Recognition Threshold Determination ^a ^

Number of Volunteers	Concentration, µg/mL					
	50	100	150	250	350	450
**1**	-	-	+	++	+++	+++++
**2**	-	+	++	+++	++++	+++++
**3**	-	+	++	++++	+++++	+++++
**4**	-	+	++	+++	+++	+++++
**5**	-	±	++	+++	++++	+++++
**6**	-	+	++	++++	++++	+++++
**7**	-	±	+	++	++++	+++++
**8**	-	+	++	++	++++	+++++

^a^ - = good, ± = tasteless, + = slightly bitter, ++ = moderate bitter, +++ = bitter, ++++ = very bitter, +++++ = awful.

**Table 5.** Taste Masking Ability of Various Formulations

Formulations	Chitosan, g	CaCl_2_/Alcl_3_, g	Taste	Concentration, µg/mL
**F1**	0.325	1.3 CaCl_2_	Good	27.71
**F2**	0.65	1.3 CaCl_2_	Good	10.70
**F3**	1.3	1.3 CaCl_2_	Good	10.44
**F4**		0.65 CaCl_2_	Good	45.44
**F5**		1.3 CaCl_2_	Good	49.43
**F6**		2.6 CaCl_2_	Good	30.91
**F’1**	0.325	1.3 AlCl_3_	Good	9.65
**F’2**	0.65	1.3 AlCl_3_	Good	10.98
**F’3**	1.3	1.3 AlCl_3_	Good	13.98
**F’4**		1.3 AlCl_3_	Good	17.71

### 4.5. *In Vitro* Release Studies

In this paper we evaluate the potential utility of natural materials, such as alginate and chitosan for taste masking of CM. This microparticulate system extends the duration period of the drug and masks the bitter taste of the drug. Alginate/chitosan particles were prepared by ionic gelation (Ca^2+^ and Al^3+^) and the effects of various factors (including chitosan and ions concentration) were examined. The ionic gelation method gave beads with a large diameter ranging from 644-1061 µm (Ca^2+^) and 912-2251 µm (Al^3+^) (Table 3). The drug encapsulation efficiency was 20.9 to 62.2% for Ca^2+^ ions with chitosan, 67.88 to 94.20% without chitosan and 47.8 to 76.1% for Al^3+^ ion with chitosan, 107.4%, without chitosan, respectively. Alginate-chitosan gels produced the egg-box structure of the beads, which gives it the spherical morphology. Figure 1A shows a microsphere prepared from adding Ca^2+^; exhibiting acceptable sphericity in all cases. The beads were off-white, transparent and elastic (Figure 1A and B).

**Figure 5.**
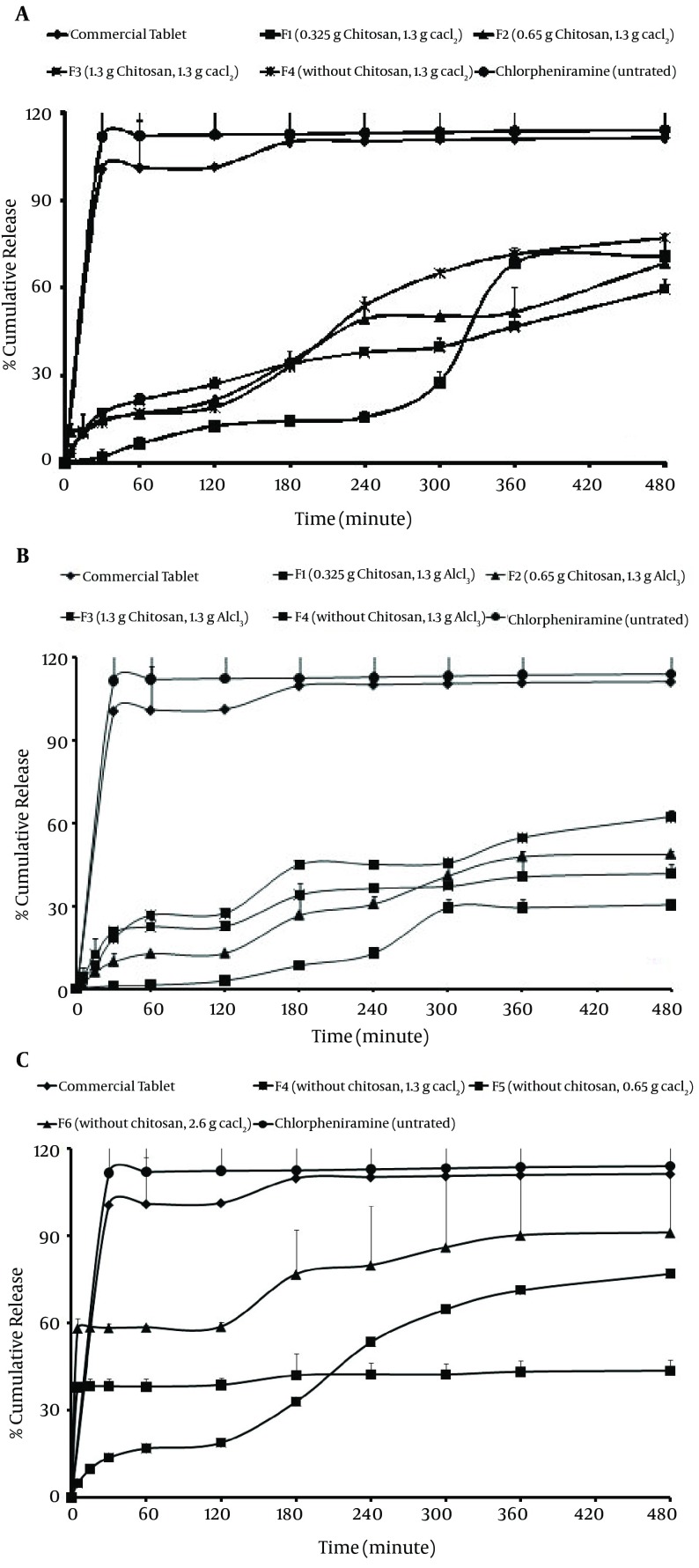
A. Cumulative Percent Release of Chlorpheniramine From Microspheres Prepared With Different Sodium Alginate:chitosan Ratios and Ion Ca^2+^/Al^3+^; B. Without Chitosan and Ion Ca^2+^; C. Untreated Chlorpheniramine and Commercial® Tablet

Figure 5A and Table 6 show that some of the microsphere formulations have a higher initial drug releases (all of formulations prepared with chitosan using Ca^2+^/Al^3+^). Initial release for F1, F2 and F3 microspheres (prepared with chitosan and Ca^2+^) within the first hour under gastric conditions was less than 10% of the total release. F1 formulation (0.325 g chitosan with Al^3+^ ion) showed the lowest burst release (1.05%) in comparison with other microsphere formulations. According to Figure 5C, F4, F5 and F6 formulations demonstrated the highest burst release (37.93%, 44.97% and 58.29%, respectively). Comparison of drug release from microspheres prepared by different ions shows that the release of drug from F4, F5 and F6 microspheres prepared using Ca^2+^ ion without chitosan is faster (t_50_% = 26.61-80.28 minutes) than the release of drug from microspheres prepared by using Al^3+^ ion without chitosan (Figure 5B and 5C). The formulation series F1, F2 and F3 contain equal amounts of Al^3+^ (Figure 5B). In these formulations, Al^3+^ decreased the drug release to a higher extent compared to formulations containing Ca^2+^.

**Table 6.** Comparison of Various Release Characteristics of Chlorpheniramine From Different Microsphere Formulations, Untreated Chlorpheniramine Powder and Commercial® Tablet a

Formulations	Chitosan, g	Cacl_2_/Alcl_3_, g	Rel_5_, %	Rel_8_, %	DE	t_50_%, min	*f*_1_
**F1**	0.325	1.3 CaCl_2_	1.99	71.81	47.30	163.82	63.64
**F2**	0.65	1.3 CaCl_2_	10.61	71.34	40.32	196.79	66.96
**F3**	1.3	1.3 CaCl_2_	3.08	59.29	36.53	184.28	70.18
**F4**		0.65 CaCl_2_	37.93	43.62	41.20	26.61	59.64
**F5**		1.3 CaCl_2_	44.97	77.08	64.19	80.28	40.98
**F6**		2.6 CaCl_2_	58.29	91.18	76.74	76	32.24
**F’1**	0.325	1.3 AlCl_3_	1.05	59.37	27.94	254.09	78.80
**F’2**	0.65	1.3 AlCl_3_	1.74	48.71	30.75	176.94	76.05
**F’3**	1.3	1.3 AlCl_3_	4.25	41.89	32.64	105.92	72.5
**F’4**		1.3 AlCl_3_	3.60	62.24	42.02	155.91	66.06
**Untreated CM**			110.99	114.06	112.38	7.08	13.39
**Commercial tablet®**			35.76	111.35	106.12	22.54	0

^a^Abbreviations: Rel, release characteristics.

## 5. Discussion

In this study we attempted to preparation of CM-loaded alginate/chitosan particulate systems by the ionic gelation method for taste masking. The percentage of encapsulation efficiency increased (up to 107%, without chitosan) as the chitosan concentration decreased.

Motwani et al. also reported that chitosan concentration had a negative effect on encapsulation efficiency because at higher concentrations, chitosan led to the formation of aggregates upon addition of alginate. Alginate and chitosan concentrations have an effect on encapsulation efficiency. Such decreases in encapsulation efficiency may be due to competition between chitosan and positively charged alginate for available acid sites on the alginate chain (2). According to Table 3, by increasing the chitosan concentration, production yield as well as encapsulation efficiency of microspheres were increased (for Ca^2+^ with chitosan) but production yield of alginate microspheres (without chitosan) was more than alginate-chitosan microspheres (58.18%) (P > 0.05). The results of the effect of the alginate/chitosan ratio on production yield, drug loading efficiency and mean particle size are shown in Table 3. This may be due to higher polymer concentration (chitosan), which increases the viscosity of the medium and makes greater availability of Ca^2+^/Al^3+^ binding sites in polymeric chains. As a result, the degree of cross-linking increased and larger droplets were formed, entrapping a greater amount of the drug. The trivalent ions cause more points of aggregation between the two contiguous alginate chains, binding them so strictly and quickly that, as a consequence, there is no time to get spherical forms, during their formation (14, 15). According to Table 3 it was found that by increasing the polymer concentration (chitosan), particle size as well as encapsulation efficiency of microspheres were increased. The loading efficiency was neither affected by the chitosan amount nor the cross-linking ion (Ca^2+^ and Al^3+^) used. Thus, this method is useful to encapsulate ionic drugs with high water solubility, such as CM. Particle size of CM beads with Ca^2+^ ion and chitosan were smaller than beads prepared with ion Al^3+^ with chitosan. The drug entrapment and drug release may be governed by the extent of surface and core cross-linking of beads, as a function of cation penetration into the bead, molecular size of the drug and valency of the cross-linking agent. Beads formed with a closely packed polymer arrangement and egg-box or three-dimensional bonding may have different drug holding and releasing abilities. The bitter taste of the drug was completely masked because of complete film formation by the -Alg and chitosan with Ca^2+^/Al^3+^ ions, which fail to release CM at salivary pH. In contrast to other techniques, such as crystallization (16), complexes with cyclodextrins (17), pH-dependent water-soluble polymers (18) and absorption to ion-exchange resin (19), the present method (ionotropic gelation) was free from any organic solvent. Recently, aqueous polymeric dispersions have played a great role in replacing organic solvents in the coating of solid dosage forms with water soluble polymers. These polymeric dispersions form a homogenous film on drying and provide a diffusion controlled release of the drug from the polymer matrix. It is important to note that the alginate gel might have acted as a barrier to the penetration of the medium, thereby suppressing the diffusion of the drug from the swollen alginate matrix. The delay in drug release is only sufficiently long enough to pass through the oral cavity, followed by complete and immediate release in gastric fluid. Chitosan, a natural polymer, was utilized for coating and incorporated in the formulation to enhance the sustaining effect of the alginate microspheres. Incorporation of chitosan in alginate microspheres affected the shape, size, surface properties and release pattern of the formulations (15). Also, Endothermic peaks of each component (chitosan, Na-Alg, CaCl_2_, AlCl_3_) wre not visible when incorporated into microsphere, whose thermogram shows only a broad and small endotherm that is probably related to dehydration and is present at a temperature of about 100˚C. CM peak might be overlapped with peaks of ions in the thermogram of microparticles. This could be ascribed to the amorphous state of the drug in the microparticles. This confirms the results obtained from DSC experiments. In summary, the FT-IR, DSC and x-ray diffraction data indicated signs of major chemical interaction between the drug and the polymer and showed that the crystallinity of the drug is reduced in the microsphere. The characteristic OH stretching, NH stretching, C-H stretching and C=O stretching of pure drug was changed in the spectra of the microspheres. It could be seen that the peaks of the complexes were shifted from those of the physical mixture. Peaks of the physical mixture appeared to be combinations of each material but they are different from those of microparticles, probably because complexation of chitosan–polyanion resulted in new chemical bonds. Chitosan peaks were similarly shifted by few cm^-1^ after complexation with alginate. Observed changes in the absorption bands of the amino groups, carboxyl groups, and amide bonds can be attributed to an ionic interaction between the carbonyl group of alginate and the amino group of chitosan. These results suggest an effective interaction between polymers and seem to be in agreement with the stoichiometric ratios between them indicating a prevalence of alginate in the final blend. Shifts on endothermic and exothermic peaks and shifts on maximum infrared peaks observed between individual polyanion complexes and final microparticle carriers were understood as ionic interactions which led to the formation of new chemical entities with different thermal and absorption properties. The results suggest that the drug maintained its chemical instability during the encapsulation process. Composition of all of the microspheres was less than the threshold bitterness value, i.e. 100 μg/mL, and completely masked the bitter taste of the drug more successfully than both polymers (Na-Alg and chitosan) (12, 13). However, a significant difference was observed between the percentages of drug released during 8 hours (Q_8_) between microspheres prepared by Ca^2+^ and Al^3+^ (P > 0.05). While release of microspheres with chitosan was lower in comparison to uncoated microspheres (without chitosan), it reached 37.93-58.29% of the total release after 5 minutes. As the coating time affects the membrane thickness, it would then be expected to have an influence on the release profile of drug encapsulated. In comparison with other microspheres, the highest drug release during 8 hours (pH = 6.8) with F6 microspheres and 2.6 g Cacl_2_ (91.18%) may be due to the higher permeability of the microspheres without chitosan. The formulation series F1, F2 and F3 contain equal amounts of Al^3+^ (Figure 5B). In these formulations, Al^3+^ decreased the drug release to a higher extent compared to formulations containing Ca^2+^. However, a significant difference was observed between the percentages of drug released during 8 hours (Q_8_) between microspheres prepared by Ca^2+^ and Al^3+^ (P > 0.05). While release of microspheres with chitosan was lower in comparison to uncoated microspheres (without chitosan), it reached 37.93-58.29% of the total release after 5 minutes. As the coating time affects the membrane thickness, it would then be expected to have an influence on the release profile of drug encapsulated. In comparison with other microspheres, the highest drug release during 8 hours (pH = 6.8) with F6 microspheres and 2.6 g Cacl_2_ (91.18%) may be due to the higher permeability of the microspheres without chitosan. The formulation series F1, F2 and F3 contain equal amounts of Al^3+^ (Figure 5B). In these formulations, Al^3+^ decreased the drug release to a higher extent compared to formulations containing Ca^2+^. The difference factor test showed that the microsphere formulation does not match the release profile of commercial tablet (Rel_8_ = 114.06, DE = 112.38, *f*_1_ = 13.39) (Table 6). As seen in Figure 5, minimal release was observed initially under gastric conditions but rapid release followed onset of simulated intestinal conditions. Al^3+^ and Ca^2+^ caused a prolonged drug release in these formulations of up to 4 hours, but after 4 hours this effect was not significant. Dave and coworkers also showed that the release of indomethacin from sustained release pellets of alginate was dependent on the concentration of Ca^2+^: a slower drug release was obtained when the concentration of Ca^2+^ increased (20). The case of the alginate/chitosan ratio is different. The presence of chitosan increases the control of release from the microsphere, since, at increasing concentration, it can form a network of bindings between the two polymer chains. This was expected, since increasing chitosan amounts in the formulations, should have increased interactions between the two polymers, forming a closer network, which should decrease the diffusion of the drug out of the bead. The reason for the burst release could be due to the presence of some CM particles close to the surface of the microspheres. When particles are prepared by ionotropic gelation without chitosan, water-soluble drugs have a tendency to migrate to the polar medium, thereby concentrating at the surface of the microspheres and inducing the burst effect (21). This potential instability may cause a part of the loaded drug to relocate at the microparticle surface, thereby become rapidly released (22).

Figure 5 also shows that in most cases a biphasic dissolution pattern was observed, when changed the pH from 1.2 to 6.8. It can be supposed that the first portion of the curves is due to CM dissolution, which starts immediately after the beginning of the test for the portion of drug very close to the surface of microspheres. After such phase, two phenomena can combine to enhance the diffusion of the remaining dispersed drug into the bulk phase and form pores within the matrix due to the initial drug dissolution which enhances the permeability of the polymer to the drug (23). The dissolution results showed that in all of formulations, the addition of Al^3+^ and Ca^2+^ had an effect on the release kinetic of CM, and the highest correlation coefficients were achieved with the Peppas model. In another study, Hosnyand coworkers showed that the release rates of diclofenac from sodium carboxymethyl cellulose and alginate beads were dependent on concentrations of the Ca^2+^ and Al^3+^ ions in the solution (24). For most formulations, high correlation was observed with the Peppas model (25, 26) (prepared with Ca^2+^) (Table 7). 

**Table 7.** Fitting Parameters of the *In vitro *Release Data to Various Release Kinetics Models ^a ^

Formulation	ORDER	MPE, %	RSQ	Slope	Intercept	K
**F1**	Peppas	14.44	0.876	0.483	-3.934	0.0196
**F2**	Square root of mass	7.44	0.97	0.001	0.044	
**F3**	Peppas	5.28	0.984	0.450	-3.410	0.0330
**F4**	Non-Conventional	1.41	0.89	0	0.071	0.0000
**F5**	Peppas	4.03	0.931	0.532	-3.854	0.0212
**F6**	Peppas	0.21	0.573	0.003	-3.410	-0.5430
**F’1**	Non-Conventional	20.37	0.954	0.001	0.003	0.0010
**F’2**	Weibull	15.40	0.961	0.757	-5.081	0.0012
**F’3**	Log-probability	11.93	0.950	0.309	-2.062	
**F’4**	Log-probability	9.12	0.976	0.441	-2.508	

^a^ Abbreviations: MPE, mean percent error; RSQ, readability strength quality.

The values of n showed that the release of CM was only controlled by diffusion, whereas in the presence of cations, the mechanism of release was slightly via erosion in F2. The n value of the commercial tablet was not calculated because the primary release percentage was more than 60%. The presence of cations was able to extend the drug release process. Bodmeier and coworkers showed that the disintegration time of alginate beads was a function of the counter ion concentration (27). This phenomenon (release kinetic) is related to an *in situ* gel formation between the cations and the anionic polymer (28). At optimum concentration, the Ca^2+^ ions are able to cross-link more efficiently with the alginate because a greater quantity of Ca^2+^ is available to bind (1.3 g Cacl_2_). Similar to our studies, Nochodchi and coworkers reported (29) that as there is more Ca^2+^ to bind, a better and stronger gel is formed around the matrix and this strong gel does not allow the dissolution medium to penetrate into the matrix at a high speed, resulting in a reduction in release rate (t50%= 163.82-196.79 minutes). In summary, CM microspheres were prepared using the iontropic gelation method. This method has been applied for the preparation of multiparticulate systems. Alginate and chitosan polymers exhibit slower rate of *in vitro* drug release initiated by lag time, which reduces the release rate of drug, as seen in conventional tablet dosage forms. In the present study, controlled release without initial peak level, achieved (very low) with these formulations, may mask the bitter taste of the drug as well as improve patient compliance.
